# Effect of Educational and Behavioral Interventions on Medication‐Overuse Headache: A Systematic Review and Meta‐Analysis

**DOI:** 10.1155/prm/3699422

**Published:** 2026-07-16

**Authors:** Pannathat Soontrapa, Prut Koonalintip, Pitchayaporn Sornplang, Benjamin R. Wakerley

**Affiliations:** ^1^ Division of Neurology, Department of Medicine, Faculty of Medicine Siriraj Hospital, Mahidol University, Bangkok, Thailand, mahidol.ac.th; ^2^ Division of Neurology, Department of Internal Medicine, Faculty of Medicine, Prince of Songkla University, Hatyai, Songkhla, Thailand, psu.ac.th; ^3^ Department of Neurology, University Hospital Birmingham, Birmingham, UK, nhs.uk; ^4^ Metabolic Neurology, Institute of Metabolism and Systems Research, College of Medical and Dental Sciences, The Medical School, The University of Birmingham, Birmingham, UK, birmingham.ac.uk

## Abstract

**Background:**

Medication‐overuse headache (MOH) is a disabling secondary headache arising from frequent acute medication intake in individuals with preexisting primary headache. Behavioral and psychosocial factors may perpetuate medication overuse. As a reduction of acute medication intake is essential for remission, educational and behavioral interventions are commonly used; however, their effectiveness remains uncertain.

**Objectives:**

To systematically evaluate the effectiveness of educational and behavioral interventions on acute medication use, headache frequency, and headache‐related disability in patients with MOH, and to assess short‐ and longer‐term outcomes compared to usual care.

**Methods:**

PubMed, Embase, and the Cochrane Central Register of Controlled Trials were searched from inception to January 2026 for randomized controlled trials evaluating educational or behavioral interventions in adults with MOH. Outcomes included reduction of acute medication use, headache frequency, and headache‐related disability. Random‐effects meta‐analyses were performed where appropriate.

**Results:**

Six trials (610 participants) were included. Brief educational interventions were associated with clinically meaningful reductions in acute medication use and headache frequency. Multiple‐session interventions, compared to minimal intervention, were associated with additional mean reductions in acute medication use of approximately 3 days per month and in headache frequency of 2.6 days per month at 3 months and 0.8 days per month at 12 months; however, between‐group differences were not statistically significant. Substantial heterogeneity was observed.

**Conclusions:**

Brief educational interventions may be considered as a potentially useful component of MOH care. Multisession interventions may offer additional benefit but remain inconclusive because of heterogeneity and limited evidence. Further high‐quality trials are needed to define optimal strategies.

## 1. Introduction

Medication‐overuse headache (MOH) arises as a consequence of frequent acute medication intake in individuals with preexisting primary headache who experience headaches on 15 or more days per month and regularly overuse acute treatments for more than 10 or 15 days per month for at least three consecutive months [1]. MOH is estimated to affect 1%–2% of people worldwide [2] and represents a major contributor to neurological disability. Together with chronic migraine, it ranks among the leading causes of years lived with disability globally [3].

The management of MOH primarily focuses on the withdrawal of overused acute medications and preventive therapies. However, behavioral and psychosocial factors may play a critical role in the development and persistence of medication overuse [4]. Although MOH shares similarities with substance use disorders, it is a distinct condition and patients should not be labeled as “addicts” [5]. Educational and behavioral interventions aim to address maladaptive beliefs and coping strategies related to acute medication use. Such interventions for pain management include patient education or counseling [6, 7], motivational interviewing and cognitive–behavioral approaches (CBT) [8, 9], relaxation techniques [10, 11], mindfulness [12, 13], and biofeedback [14].

Medication overuse represents the defining and perpetuating mechanism of MOH, and its reduction is essential for clinical remission [15]. Although educational and behavioral interventions differ in format and intensity, they share a common objective: modifying acute medication–taking behavior. Thus, despite clinical variability in delivery, these interventions are conceptually unified by their focus on reducing medication overuse.

Several studies have evaluated educational and behavioral interventions in MOH. Brief outpatient advice has shown benefit in uncomplicated cases [16, 17], and more recent randomized trials of multisession interventions suggest potential efficacy, although effect sizes remain inconsistent [18, 19]. Overall, their effectiveness and role in routine practice remain uncertain.

We conducted a systematic review and meta‐analysis to evaluate the effects of educational and behavioral interventions on acute medication use, headache frequency, and disability in MOH, compared to usual care or control, at postintervention and 1‐year follow‐up.

## 2. Method

This systematic review was conducted and reported in accordance with the Preferred Reporting Items for Systematic Reviews and Meta‐Analyses (PRISMA) 2020 statement [20]. The protocol for this systematic review was prospectively registered in the International Prospective Register of Systematic Reviews (PROSPERO) (registration number: CRD420251275673).

### 2.1. Eligibility Criteria

Eligibility criteria were established using the population, intervention, comparison, outcomes, and study design (PICOS) framework. We included randomized controlled trials enrolling adults (≥ 18 years) with MOH diagnosed according to the International Classification of Headache Disorders (ICHD‐2, ICHD‐3 beta, or ICHD‐3) [1, 21, 22].

Only RCTs were eligible for inclusion. Interventions of interest comprised patient education, brief advice or counseling, motivational interviewing, CBT, mindfulness‐based interventions, relaxation therapy, and biofeedback. These interventions were delivered either as standalone strategies or as adjuncts to pharmacological management and were compared to no or minimal intervention or usual care.

Outcomes were acute medication use (days per month), headache frequency (days per month), and headache‐related disability assessed using validated instruments. Outcome time points were defined as the end of the intervention period (3 months ± 1 month) and/or approximately 1 year of follow‐up (12 months ± 3 months) to evaluate the sustainability of effects [23].

Studies were excluded if relevant outcome data could not be extracted or if the independent effect of the educational or behavioral intervention could not be assessed (e.g., education alone versus pharmacological treatment alone).

### 2.2. Information Sources and Search Strategy

A systematic search of PubMed, Embase (Ovid), and the Cochrane Central Register of Controlled Trials was conducted from database inception to January 2026 to identify English‐language randomized controlled trials. We used keywords relating to MOH, patient education, motivational interviewing, CBT, mindfulness‐based interventions, relaxation therapy, biofeedback, and related synonyms. Two reviewers (PS and PK) independently screened the titles and abstracts of all retrieved records using Rayyan [24] to identify potentially eligible studies. Potentially eligible studies underwent independent full‐text review by PS and PK to determine eligibility based on the predefined inclusion and exclusion criteria. Disagreements were resolved by consultation with a third reviewer (PSo). The complete, detailed search syntax for each database can be found in Supporting Methods S1: Search syntax.

### 2.3. Data Collection Process

Two reviewers (PS and PK) independently extracted data on study characteristics, participant demographics, intervention and comparator details, and outcome measures, including mean values and standard deviations (SDs) for acute medication use, headache frequency, and headache‐related disability. Disagreements were resolved through discussion with a third reviewer (PSo).

We anticipated variability in outcome assessment across studies, particularly with respect to differences in the timing of outcome measurement at the end of the intervention period (3 months ± 1 month) and during follow‐up for sustainability of effects (12 months ± 3 months). To address this heterogeneity, outcome time points were standardized to 3 months and 12 months, respectively, wherever possible.

Outcomes were measured on continuous scales and reported as mean reductions from baseline with corresponding SDs. When required data were missing, we derived the necessary values from available information, such as calculating SD from reported 95% confidence intervals (95% CIs) and sample sizes. SDs of change scores were calculated from baseline and follow‐up SDs assuming a correlation coefficient of 0.5, in accordance with the Cochrane Handbook [25]. When data could not be derived, the study authors were contacted for clarification.

### 2.4. Risk of Bias (RoB) Assessment

PS and PK independently assessed the RoB of the included RCTs using the Cochrane RoB 2 tool [26]. Each study was evaluated across five domains. The overall RoB was judged as low risk, some concerns, or high risk. Disagreements were resolved through discussion with a third reviewer (PSo). Risk‐of‐bias plots were generated using the robvis tool [27].

### 2.5. Data Synthesis and Statistical Analysis

Mean differences (MDs) with corresponding SDs were used as effect measures. Meta‐analyses were performed when at least three studies reported comparable outcomes. Separate analyses were performed for outcomes assessed at the end of the intervention period and during follow‐up (approximately 1 year) to evaluate the sustainability of effects [23]. Random‐effects models were applied to account for anticipated clinical and methodological heterogeneity. All statistical analyses were conducted using R software, with meta‐analysis performed via the meta package [28]. The results of the pooled syntheses were presented using forest plots. Statistical heterogeneity was considered significant when the *I* ^2^ value was ≥ 50% or when the τ^2^ estimate exceeded 0.1 [29]. To explore potential sources of heterogeneity, we performed sensitivity and leave‐one‐out sensitivity analyses, whereby each study was sequentially omitted, and the pooled effect and heterogeneity estimates were re‐calculated. When meta‐analysis was not feasible, results were summarized narratively and presented in structured tables. The certainty of evidence for each outcome was assessed using the GRADE approach [30]. Evidence was evaluated across five domains: RoB, inconsistency, indirectness, imprecision, and publication bias. The certainty of evidence was rated as high, moderate, low, or very low.

## 3. Results

### 3.1. Study Selection

**FIGURE 1:**
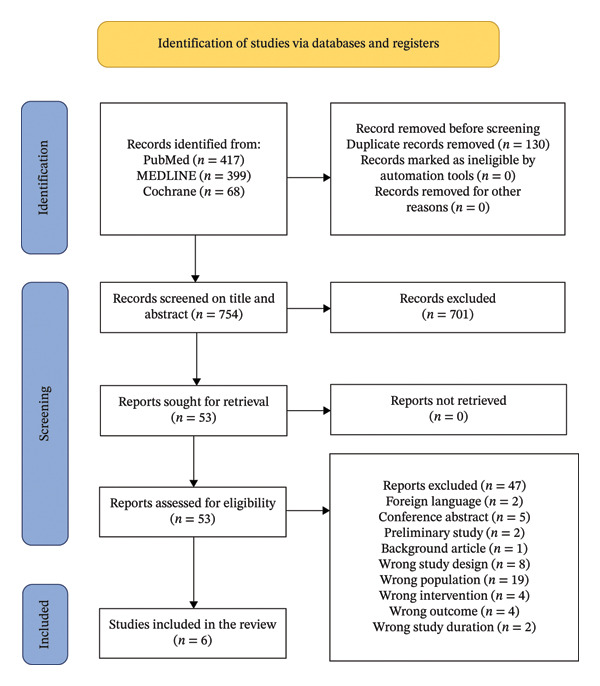
PRISMA flow diagram of the study selection process.

We identified a total of 884 studies in the initial search. After removing duplicates, 754 records remained for title and abstract screening. After the initial screening, 53 studies were identified for full‐text screening. Forty‐seven studies were excluded through full‐text review, leaving 6 RCTs for final analyses. The flow diagram is presented in Figure 1.

### 3.2. Study Characteristics

The included trials were published between 2015 and 2023 and were conducted across outpatient and specialist headache clinic settings with a total of 610 subjects. Sample sizes ranged from 27 to 179 participants. All studies enrolled adult patients diagnosed with MOH according to the ICHD criteria [1, 21, 22]. The mean age of participants was approximately 45 years (ranged 40–51 years), and the study populations were predominantly female (approximately 82% across trials). Chronic migraine was the predominant primary headache disorder, accounting for nearly all participants across the included studies.

Among the six included trials, two evaluated single‐session brief educational interventions [31, 32], whereas four assessed multiple‐session interventions, including electromyographic (EMG) biofeedback [33], patient education program [34], CBT and motivational interview [18], and mindfulness‐based intervention [19]. Interventions were delivered either as standalone strategies or as adjuncts to pharmacological management and were compared to usual care or minimal intervention. Follow‐up durations ranged from the end of the intervention period (2–4 months) to assessments of sustainability at longer‐term follow‐up (9–12 months). Detailed baseline characteristics of the included studies are summarized in Table 1.

**TABLE 1:** Baseline information of the enrolled studies.

Study	Sample size	Intervention	Control	Duration of intervention	On headache prevention	Outcome	Follow‐up
Kristoffersen et al. [31]	60 (24, 36)	Brief intervention (5 questions by GP)	Business as usual	Single	13 vs. 19%	1. Acute medication use 2. Headache frequency 3. Headache index	3 months
Moraes Alves [32]	69 (37, 32)	Structured counseling (FRAMES)	Unstructured counseling	Single	97.3 vs. 96.8%	1. Acute medication use 2. Headache frequency 3. HIT‐6	2 months
Rausa et al. [33]	27 (15, 12)	EMG biofeedback	Control group	9 weekly sessions	100 vs. 100%	1. Acute medication use 2. Headache frequency	9 weeks
Mose et al. [34]	98 (48, 50)	Educational program (individual or group sessions 1–1.5 h by a nurse and a physiotherapist)	Control group	6 sessions within 12 weeks	96 vs. 92%	1. Acute medication use 2. Headache frequency 3. MIDAS	4 and 9 months
Pijpers et al. [18]	179 (90, 89)	CBT and motivational interviewing (a 30‐min consultation by a headache nurse followed by telephone follow‐up)	Outpatient standard treatment	At least 3 follow‐up telephonic contacts (every 2–4 weeks) Within 12 weeks	BTX‐A 50 vs. 50%	1. Acute medication use 2. Headache frequency	3 and 12 months
Grazzi et al. [19]	177 (88, 89)	Mindfulness (group sessions of mindfulness practice, 90 min by mindfulness instructor)	Treatment as usual	6 weekly sessions	NA	1. Acute medication use 2. Headache frequency 3. HIT‐6, MIDAS	3 and 12 months

*Note:* BTX‐A, Botulinum toxin type A; EMG, electromyography; FRAMES, feedback about personal risk, responsibility of patient, advice to change, menu of options, empathy, and self‐efficacy enhancement; MIDAS, migraine disability assessment.

Abbreviations: CBT, cognitive–behavioral therapy; HIT‐6, Headache impact test–6; NA, not available.

### 3.3. RoB

Risk‐of‐bias assessments for the six included randomized controlled trials using the Cochrane RoB 2 tool are summarized in Supporting Figure S1. Overall, one trial was judged to be at low RoB [18], three trials were judged as having some concerns [19, 31, 34], and two trials were judged to be at high RoB [32, 33].

The most frequent source of concern was bias due to deviations from intended interventions (Domain 2), reflecting the inherent challenges of blinding participants and personnel in educational and behavioral interventions. High RoB was identified in the domain of missing outcome data (Domain 3) in two studies, which contributed to their overall high risk‐of‐bias judgment.

Overall, the risk‐of‐bias assessment indicated moderate methodological quality among the included studies, with limitations mainly related to deviations from intended interventions, particularly challenges in blinding, and incomplete outcome data.

### 3.4. Effects of Brief Educational Interventions

Two RCTs evaluated single‐session brief educational interventions (Table 2). These interventions were delivered in outpatient settings and focused on providing advice regarding medication overuse and headache management. Data on the longer‐term sustainability of effects were not available from either trial.

**TABLE 2:** Reduction from baseline in acute medication use, headache frequency, and headache‐related disability following brief educational interventions in medication‐overuse headache.

Study	*N*	Reduction of acute medication use (days per month), mean (SD)		Reduction of headache frequency (days per month), mean (SD)		Reduction of HIT‐6, mean (SD)		Follow‐up
	I vs C	I	C	I	C	I	C	
Kristoffersen et al. [31]	24 vs. 36	10.4 (9.4)	0.6 (7.1)	7.4 (8.5)	0.6 (5.4)	NA	NA	3 months
Moraes Alves [32]	37 vs. 32	18.1 (6.6)	17.9 (8.1)	11.8 (8.5)	9.3 (8.6)	7.7 (9.1)	6.4 (10.2)	2 months

*Note:* C, control; I, intervention.

Abbreviations: HIT‐6, Headache impact test–6; SD, standard deviation.

In the trial by Kristoffersen et al. [31], a brief educational intervention was superior to usual care (no intervention) at the end of the intervention period (3 months), without the use of additional pharmacotherapy. Participants receiving the intervention demonstrated a substantially greater reduction in acute medication use, with a mean reduction of 10.4 days per month, compared to a reduction of 0.6 days per month in the control group (10.4 ± 9.4 vs. 0.6 ± 7.1 days/month). A similar pattern was observed for headache frequency, with greater reductions in the intervention group than in the control group (7.4 ± 8.5 vs. 0.6 ± 5.4 days per month).

In a trial evaluating FRAMES‐based interventions [32], structured and unstructured brief educational interventions demonstrated comparable effectiveness and were associated with substantial reductions in the outcomes. Both approaches were associated with similar mean reductions in acute medication use (intervention vs. control: 18.1 ± 6.6 vs. 17.9 ± 8.1 days per month) and headache frequency (11.8 ± 8.5 vs. 9.3 ± 8.6 days per month), as well as comparable improvements in headache‐related disability measured by the Headache impact test–6 (HIT‐6) [35] (7.7 ± 9.1 vs. 6.4 ± 10.2).

### 3.5. Effects of Multiple‐Session Educational and Behavioral Interventions

Four RCTs evaluated the effects of multiple‐session educational and/or behavioral interventions in patients with MOH, including EMG biofeedback [33], patient education program [34], CBT and motivational interview [18], and mindfulness‐based intervention [19]. All included studies reported outcomes at the end of the intervention period (approximately 3 months), while three out of four studies additionally reported outcomes at approximately 12 months to assess the sustainability of effects.

### 3.6. Acute Medication Use Reduction (Figure 2)

#### 3.6.1. End of Intervention Period (Approximately 3 months)

Four studies involving 481 participants evaluated acute medication use reduction. Educational and behavioral interventions were associated with a greater reduction in acute medication use than control, with a MD of 3.1 days per month; however, the between‐group difference did not reach statistical significance (MD: 3.1 days/month, 95% CI: −0.3 to 6.5). Substantial heterogeneity was observed across studies (*I* ^2^ = 70.9%, *τ* ^2^ = 7.6).

**FIGURE 2:**
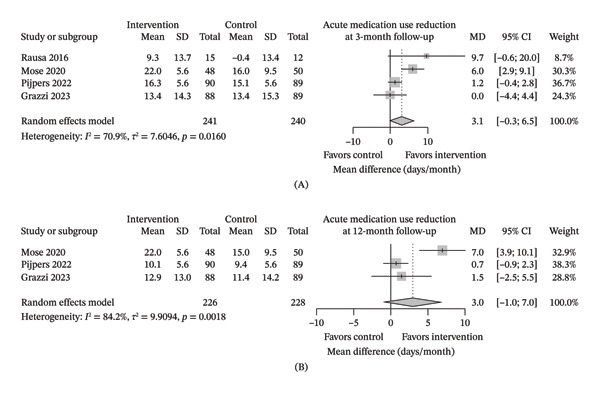
Forest plot of the effect of interventions on acute medication use reduction at 3‐month (A) and 12‐month (B) follow‐up, compared to control. CI: confidence interval; *I* ^2^ and *τ* ^2^: heterogeneity statistic; MD: mean difference; SD: standard deviation.

#### 3.6.2. Sustainability of Effects (Approximately 12 months)

Three trials (*n* = 454) reported outcomes at approximately 12 months. The pooled effect estimate suggested a trend toward greater reduction in acute medication use with educational and behavioral interventions, but the difference was not statistically significant (MD: 3 days/month, 95% CI: −0.1 to 7.0). Between‐study heterogeneity was considerable (*I* ^2^ = 84.2%, *τ* ^2^ = 9.9), indicating substantial variability in longer‐term effects.

### 3.7. Headache Frequency Reduction (Figure 3)

**FIGURE 3:**
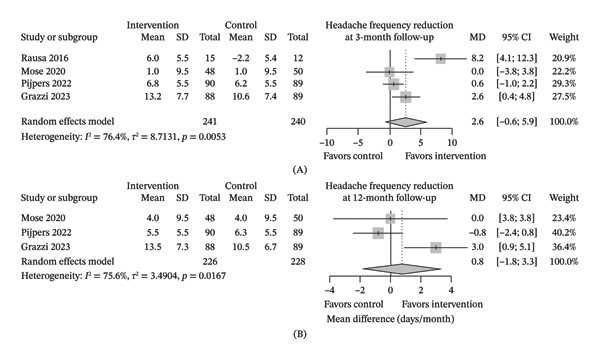
Forest plot of the effect of interventions on headache frequency reduction at 3‐month (A) and 12‐month (B) follow‐up, compared to control. CI: confidence interval; *I* ^2^ and *τ* ^2^: heterogeneity statistic; MD: mean difference; SD: standard deviation.

#### 3.7.1. End of Intervention Period (Approximately 3 Months)

Four trials including 481 participants assessed headache frequency reduction at around 3 months. Educational and behavioral interventions were associated with a greater reduction in headache frequency than control, with a MD of 2.6 days per month; however, the between‐group difference did not reach statistical significance (MD: 2.6 days/month, 95% CI: −0.6 to 5.9). Heterogeneity was high (*I* ^2^ = 76.4%, *τ* ^2^ = 8.7), reflecting differences in intervention intensity and outcome measurement across studies.

#### 3.7.2. Sustainability of Effects (Approximately 12 months)

Three studies (*n* = 454) contributed data on headache frequency at approximately 12 months. The pooled estimate showed no clear sustained benefit of intervention over control (MD: 0.8 days/month, 95% CI: −1.8 to 3.3), with substantial heterogeneity across studies (*I* ^2^ = 75.6%, *τ* ^2^ = 3.5).

### 3.8. Headache‐Related Disability

Two RCTs evaluated the effects of multiple‐session educational and behavioral interventions on headache‐related disability, assessed using the Migraine Disability Assessment (MIDAS) score and HIT‐6. Meta‐analysis was not feasible because of the limited number of trials.

In the trial by Mose et al. [34], headache‐related disability improved over time in both the intervention and control groups. At 4 months, the mean reduction in MIDAS score was 5.7 ± 58.2 in the intervention group (*n* = 48) and 4.6 ± 56.7 in the control group (*n* = 50). At 9 months, further reductions were observed, with mean reduction MIDAS scores of 10.6 ± 59.2 in the intervention group and 15.9 ± 53.4 in the control group. However, no statistically significant between‐group differences were reported at either time point.

In the mindfulness‐based intervention trial [19], headache‐related disability was assessed using both the HIT‐6 and MIDAS instruments. The authors reported that participants receiving the intervention achieved greater improvements in MIDAS score compared to treatment as usual, but this between‐group difference was evident only at the 12‐month follow‐up (mean reduction ± SD: intervention 46.8 ± 44.7 vs. control 23.5 ± 53.3), whereas no significant between‐group differences were observed for either MIDAS at earlier time points or for HIT‐6.

### 3.9. Certainty of Evidence

The certainty of evidence was rated as very low for all evaluated outcomes according to the GRADE approach [30], mainly because of RoB, inconsistency, and imprecision (Supporting Table S1).

### 3.10. Sensitivity Analysis

#### 3.10.1. Acute Medication Use

Sensitivity analyses excluding the trial at high RoB did not change the pooled estimate for acute medication use at 3 months (MD: 2.5 days/month, 95% CI: −1 to 5.9), and substantial heterogeneity persisted (*I* ^2^ = 75.7%; Supporting Figure S2). Leave‐one‐out analyses showed that omission of Mose et al. [34] attenuated the pooled effect and substantially reduced heterogeneity at 3 months (*I* ^2^ from 70.9% to 31.4%), while eliminating heterogeneity entirely at 12 months (*I* ^2^ = 0%). In contrast, omission of the other trials had little impact on the overall findings (Supporting Figure S3), indicating inconsistency of effects across studies.

### 3.11. Headache Frequency

Sensitivity analyses excluding the trial at high RoB did not materially alter the pooled estimate for headache frequency reduction at 3 months. The intervention remained associated with a small, nonsignificant reduction in headache days compared to control (MD: 1.2 days per month, 95% CI: −0.3 to 2.7), with low heterogeneity (*I* ^2^ = 17.9%; Supporting Figure S4), suggesting that RoB contributed to between‐study heterogeneity.

Leave‐one‐out analyses for headache frequency at 12 months showed that omission of Grazzi et al. [19] eliminated heterogeneity (*I* ^2^ = 0%) and shifted the pooled estimate toward the null and in the opposite direction (MD: −0.7 days/month, 95% CI: −2.1 to 0.8), whereas omission of the other trials had little impact on heterogeneity (*I* ^2^ > 60%) (in Supporting Figure S5), indicating substantial inconsistency in treatment effects across studies.

## 4. Discussion

The management of MOH focuses on withdrawal and preventive therapy; however, the role of educational and behavioral interventions remains uncertain [36]. This systematic review and meta‐analysis synthesizes RCT evidence on such interventions in MOH. Despite variations in format and intensity, these approaches share a common aim: reducing acute medication overuse, the key behavioral driver of MOH, across short‐ and longer‐term follow‐up.

### 4.1. Brief Educational Interventions

Our results suggest that brief educational interventions were associated with clinically meaningful improvements in acute medication use, headache frequency, and headache‐related disability in patients with MOH, across both structured and unstructured counseling approaches. These findings indicate that even minimal educational input may be sufficient to trigger behavioral change in some patients. They also support the concept that raising awareness of medication overuse and reinforcing appropriate use of preventive strategies can positively influence patient behavior [17, 37].

A brief educational intervention delivered to patients with uncomplicated MOH in a primary‐care setting was associated with substantial reductions in both acute medication use (10.4 days per month) and headache frequency (7.4 days per month) at 3 months [31]. These effects are likely attributable primarily to the educational intervention itself, as only a small proportion of participants received preventive pharmacotherapy (13% and 19% in the intervention and control groups, respectively), supporting the effectiveness of simple advice and education in this population [17, 38].

Both structured counseling and unstructured counseling were associated with substantial reductions in acute medication use (approximately 18 days per month) and headache frequency (approximately 9.3–11.8 days per month) [32]. However, the larger magnitude of improvement compared to the BIMOH study [31] may partly reflect the high uptake of preventive pharmacotherapy, about 97% (97.3% vs 96.8%), supporting a synergistic benefit of combining strategies [36, 39].

Importantly, a subsequent long‐term follow‐up of the BIMOH study [40] reported sustained reductions in headache frequency and medication use over a mean follow‐up of 16 months, with a relapse rate of only 8.3%. Although this study was not included in the present quantitative synthesis because it exceeded the prespecified follow‐up period, it supports the potential long‐term durability of brief educational interventions.

These findings should be interpreted with caution, as the number of participants was small and the certainty of evidence was limited by the high RoB, particularly due to missing outcome data. Larger, methodologically robust trials are therefore needed to confirm the magnitude and durability of these effects. Nevertheless, given their simplicity, low cost, and absence of apparent harms [41, 42], brief educational interventions could reasonably be offered to all patients with MOH as part of routine care [43].

### 4.2. Multiple‐Session Educational and Behavioral Interventions

Our findings suggest that, compared to minimal intervention (mostly brief education at the initial visit), multiple‐session educational and behavioral interventions were associated with mean reductions in acute medication use of approximately 3 days per month at both the end of the intervention period and at longer‐term follow‐up. Corresponding reductions in headache frequency were approximately 2.6 days per month at 3 months and 0.8 days per month at 12 months. However, none of these between‐group differences reached statistical significance, and substantial heterogeneity reflected inconsistent treatment effects across studies in the sensitivity analysis.

To our knowledge, no prior meta‐analysis has quantified reductions in acute medication use and headache frequency following multisession educational and behavioral interventions in MOH. In primary headache populations, such interventions typically yield modest reductions in headache frequency (approximately 0.7–1 day per month). [42, 44–46]. In contrast, our meta‐analysis observed a larger short‐term improvement, with headache frequency reduced by 2.6 days per month at 3 months. One plausible explanation is that the observed benefit reflects two synergistic mechanisms: (1) a direct effect of the intervention on headache‐related behaviors and coping (e.g., stress management, sleep, and trigger control) [47, 48] and (2) an indirect effect mediated by reduced acute medication use, which is itself a key driver of headache in MOH [49, 50].

Interestingly, although reductions in acute medication use were of similar magnitude in both the short and longer term (approximately 3 days per month), the reduction in headache frequency was more pronounced in the short term (2.6 days per month) and attenuated over time, reaching only 0.8 days per month at 12 months. This pattern supports that acute medication use is not the sole determinant of headache burden in MOH. Rather, the pathophysiology of MOH is complex and likely influenced by multiple interacting factors. In addition to medication overuse, genetic predisposition [51] may interact with biological, behavioral [52, 53], and psychological factors [54] to shape the development, mechanisms, and clinical course of MOH [15]. Another possible explanation is that substantial reversion of MOH in both groups may have led to parallel headache frequency reductions [55]. This was accompanied by reductions in headache frequency in both arms, leading to diminishing between‐group differences over time. In addition, Hawthorne effects [56], whereby participants modify their behavior because they are aware of being observed in a research setting, may have contributed to improvements in both intervention and control groups over time. Such research participation effects have been described across a range of clinical studies and may attenuate between‐group differences during follow‐up.

The substantial heterogeneity observed is likely explained by the diversity of the included interventions, which ranged from EMG biofeedback [33] to patient education programs [34], CBT and motivational interviewing [18], and mindfulness‐based approaches [19]. Previous meta‐analyses of behavioral interventions for migraine prevention indicate that CBT, relaxation training, and mindfulness‐based therapies are each associated with small but clinically meaningful reductions in headache frequency (around 1 day per month), whereas evidence for biofeedback and acceptance and commitment therapy remains inconclusive [42]. Importantly, this heterogeneity does not imply that all multisession interventions for MOH are ineffective. Rather, pooling interventions with varying efficacy, intensity, and modes of delivery may have diluted the overall effect. The pooled estimates should therefore be interpreted with caution, as the study by Mose et al. appeared to contribute substantially to the observed heterogeneity.

Unfortunately, meta‐analysis of headache‐related disability was not feasible because of the limited number of trials. Therefore, the benefit remains inconclusive.

Overall, multiple‐session educational and behavioral interventions should be considered a management option for patients with MOH. These interventions appear to be safe, but their implementation in routine clinical practice may be challenging, as they require structured programs, trained personnel, and often multidisciplinary or nurse‐led support to ensure adherence and continuity of care.

### 4.3. Strength and Limitations

This systematic review and meta‐analysis provides a comprehensive synthesis of RCTs evaluating brief and multisession educational and behavioral interventions in MOH, enabling unified comparison of commonly used approaches. Our findings offer clinically relevant insights into the magnitude and durability of treatment effects.

Educational and behavioral interventions may remain important components of MOH management. Although emerging evidence suggests that anti‐CGRP therapies may achieve remission without formal withdrawal [57], behavioral interventions may provide complementary benefits through improved self‐management and relapse prevention. Furthermore, these approaches may be particularly valuable in resource‐limited settings, where access to advanced migraine therapies remains restricted by availability or cost.

Several limitations should be acknowledged. First, the certainty of evidence was rated as very low for all evaluated outcomes according to the GRADE approach [30], primarily because of RoB, inconsistency, and imprecision. This reflected the small number of available trials and methodological concerns across several studies. In addition, challenges inherent to behavioral intervention trials, particularly difficulties in blinding participants and personnel, should be considered when interpreting the findings.

Second, substantial heterogeneity likely reflects the diversity of multisession interventions. Acute medication overuse may contribute to MOH through behavioral and psychological mechanisms, sometimes overlapping with features of substance use disorder [58]. Educational and behavioral interventions share a common goal: to enhance patients’ awareness of MOH and to promote strategies that reduce reliance on acute medications, thereby targeting a key perpetuating mechanism of the disorder. On this basis, we considered these interventions sufficiently conceptually similar to be grouped together in the meta‐analysis; however, their specific components and intensity vary, and their direct effects on headache‐related behaviors and coping may therefore differ. Furthermore, the relatively small number of included studies and participants may have limited the statistical power of the meta‐analysis to detect modest but clinically meaningful between‐group differences.

Moreover, the proportion of the use of preventive pharmacotherapy and underlying primary headache subtypes varied across trials, which may have contributed to between‐study heterogeneity. However, as all included studies were RCTs, baseline characteristics and preventive treatment use were generally balanced between intervention and control groups within each study, supporting that the observed effects are likely attributable to the interventions.

Finally, for some outcomes, numerical data were not fully extractable. In particular, SD for acute medication use and headache frequency at 3 and 12 months were not reported in the study by Pijper et al. [18], and despite contacting the author, these data were unavailable; baseline SD were therefore used as a proxy [25], an approach that is methodologically acceptable. Conversely, we obtained additional data directly from the author of the MIND‐CM study [19] to improve data accuracy, but these data were reported over 3‐month intervals and required conversion to monthly units, which may have introduced additional uncertainty.

## 5. Conclusion

Brief educational interventions were associated with clinically meaningful reductions in acute medication use, headache frequency, and headache‐related disability in patients with MOH and may be considered as a potentially useful component of MOH management. Multisession educational and behavioral interventions were associated with additional mean reductions in acute medication use of approximately 3 days per month at both short‐ and longer‐term follow‐up, and in headache frequency of approximately 2.6 days per month at 3 months and 0.8 days per month at 12 months, although these between‐group differences did not reach statistical significance. These interventions may therefore be considered an option for MOH management in settings where such services are available. Overall, educational and behavioral strategies appear to be safe, low‐risk, and potentially effective adjuncts to the management of MOH, but further well‐designed trials are needed to define the most effective components and optimal delivery models.

**Nomenclature**
CBT: Cognitive behavioral therapy
CI: Confidence interval
EMG: Electromyographic
FRAMES: Feedback, responsibility, advice, menu of options, empathy, and self‐efficacy
HIT‐6: Headache impact test–6
ICHD: International Classification of Headache Disorders
MD: Mean difference
MIDAS: Migraine Disability Assessment
MOH: Medication‐overuse headache
PRISMA: Preferred Reporting Items for Systematic Reviews and Meta‐analyses
PROSPERO: International Prospective Register of Systematic Reviews
RCT: Randomized controlled trial
RoB 2: Risk of Bias 2 tool
SD: Standard deviation

## Author Contributions

Benjamin R. Wakerley initiated the research project, critically reviewed the manuscript, and approved the final version. Pitchayaporn Sornplang served as the first reviewer and drafted the initial version of the manuscript. Prut Koonalintip acted as the second reviewer, contributed to the conception and design of the study, contacted authors to obtain raw data, analyzed the data, and edited the manuscript. Pannathat Soontrapa served as the third reviewer and edited the manuscript.

## Funding

No funding was received for this research.

## Conflicts of Interest

Benjamin R. Wakerley is the founder of Ceftronics Limited and the CEFREF migraine mobile application. He has performed consultancy for Invex Therapeutics and received honoraria from AbbVie. The remaining authors declare no conflicts of interest.

## Supporting Information

Additional supporting information can be found online in the Supporting Information section.

## Supporting information


**Supporting Information 1** Supporting Methods S1 describes the detailed search strategies used in PubMed, Embase (Ovid), and the Cochrane Library for identifying randomized controlled trials evaluating educational and behavioral interventions for medication‐overuse headache. Supporting Figure S1 shows the RoB assessment of the included studies. Supporting Figure S2 presents a sensitivity analysis excluding a trial at high RoB, showing a forest plot of the effect of interventions on acute medication use reduction at 3‐month follow‐up compared to control. Supporting Figure S3 shows leave‐one‐out sensitivity analyses of the effect of interventions on acute medication use reduction at 3‐month and 12‐month follow‐up. Supporting Figure S4 presents a sensitivity analysis excluding trials at high RoB, showing a forest plot of the effect of interventions on headache frequency reduction at 3‐month follow‐up compared to control. Supporting Figure S5 shows leave‐one‐out sensitivity analyses of the effect of interventions on headache frequency reduction at 3‐month and 12‐month follow‐up. Supporting Table S1: GRADE assessment of the certainty of evidence for acute medication use reduction and headache frequency reduction at 3‐ and 12‐month follow‐up.

**Supporting Information 2** PRISMA_2020_checklist.

## Data Availability

The data that support the findings of this study are available in the Supporting Information of this article.
