# Supplementary material for: Effect of Educational and Behavioral Interventions on Medication‐Overuse Headache: A Systematic Review and Meta‐Analysis
Source: Pain Res Manag. 2026 Jul 16;2026:3699422. doi: 10.1155/prm/3699422 (PMC13373701; doi:10.1155/prm/3699422)
Supplement: Supplementary file 1 — Supporting Information 1 Supporting Methods S1 describes the detailed search strategies used in PubMed, Embase (Ovid), and the Cochrane Library for identifying randomized controlled trials evaluating educational and behavioral interventions for medication‐overuse headache. Supporting Figure S1 shows the RoB assessment of the included studies. Supporting Figure S2 presents a sensitivity analysis excluding a trial at high RoB, showing a forest plot of the effect of interventions on acute medication use reduction at 3‐month follow‐up compared to control. Supporting Figure S3 shows leave‐one‐out sensitivity analyses of the effect of interventions on acute medication use reduction at 3‐month and 12‐month follow‐up. Supporting Figure S4 presents a sensitivity analysis excluding trials at high RoB, showing a forest plot of the effect of interventions on headache frequency reduction at 3‐month follow‐up compared to control. Supporting Figure S5 shows leave‐one‐out sensitivity analyses of the effect of interventions on headache frequency reduction at 3‐month and 12‐month follow‐up. Supporting Table S1: GRADE assessment of the certainty of evidence for acute medication use reduction and headache frequency reduction at 3‐ and 12‐month follow‐up. [file PRM-2026-3699422-s002.docx]

**Supplemental Methods S1: Search syntax**

**Pubmed**

1. “Medication-overuse headache” OR "medication overuse" OR “rebound headache”

2. “brief intervention” OR “client education” OR “patient education” OR “educational programs” OR counseling OR “educational counseling”

3. “behavioral intervention” OR “behavior modification” OR “cognitive behavior therapy” OR “cognitive therapy” OR “coping behavior” OR “problem solving” OR psychotherapy

4. “biofeedback training” OR biofeedback OR neurofeedback

5. “relaxation therapy” OR “relaxation” OR “muscle relaxation” OR “breathing exercise”

6. meditation OR mindfulness OR “mindfulness- based interventions”

7. “motivational interviewing” OR “Stages of Change”

8. “Group Psychotherapy” OR “support groups” OR “group counseling”

9. “randomized controlled trial” OR “clinical trial”

1. AND (2 OR 3 OR 4 OR 5 OR 6 OR 7 OR 8) AND 9

("Medication overuse headache" OR "Medication overuse" OR “rebound headache”) AND ((“brief intervention” OR “client education” OR “patient education” OR “educational programs” OR counseling OR “educational counseling”) OR (“behavioral intervention” OR “behavior modification” OR “cognitive behavior therapy” OR “cognitive therapy” OR “coping behavior” OR “problem solving” OR psychotherapy) OR (“biofeedback training” OR biofeedback OR neurofeedback) OR (“relaxation therapy” OR “relaxation” OR “muscle relaxation” OR “breathing exercise”) OR (meditation OR mindfulness OR “mindfulness- based interventions”) OR (“motivational interviewing” OR “Stages of Change” ) OR (“Group Psychotherapy” OR “support groups” OR “group counseling”)) AND (“randomized controlled trial” OR “clinical trial”) ("Medication overuse headache" OR "Medication overuse" OR “rebound headache”) AND ((“brief intervention” OR “client education” OR “patient education” OR “educational programs” OR counseling OR “educational counseling”) OR (“behavioral intervention” OR “behavior modification” OR “cognitive behavior therapy” OR “cognitive therapy” OR “coping behavior” OR “problem solving” OR psychotherapy) OR (“biofeedback training” OR biofeedback OR neurofeedback) OR (“relaxation therapy” OR “relaxation” OR “muscle relaxation” OR “breathing exercise”) OR (meditation OR mindfulness OR “mindfulness- based interventions”) OR (“motivational interviewing” OR “Stages of Change” ) OR (“Group Psychotherapy” OR “support groups” OR “group counseling”)) AND (“randomized controlled trial” OR “clinical trial”)

**Embase (OVID)**

(medication overuse headache/OR ("medication-overuse headache" OR "medication overuse" OR "rebound headache").ti,ab.)

AND

(patient education/ OR health education/ OR behavior therapy/ OR cognitive therapy/ OR psychotherapy/ OR biofeedback/ OR relaxation therapy/ OR mindfulness/ OR ("brief intervention" OR "client education" OR "patient education" OR "educational program*" OR counseling OR "educational counseling" OR "behavioral intervention" OR "behavior modification" OR "cognitive behavioral therapy" OR "cognitive behaviour therapy" OR CBT OR "cognitive therapy" OR psychotherapy OR biofeedback OR neurofeedback OR relaxation OR "muscle relaxation" OR "breathing exercise*" OR meditation OR mindfulness OR "mindfulness-based intervention*" OR "motivational interviewing" OR "stages of change" OR "group psychotherapy" OR "support group*" OR "group counseling").ti,ab.)

**Cochranes**

#1 ("medication overuse headache"):ti,ab,kw (Word variations have been searched)

#2 "medication overuse" OR "rebound headache"

#3 #1 OR #2

#4 "brief intervention" OR "patient education" OR "client education" OR "educational program" OR

counseling OR "educational counseling" OR "behavioral intervention" OR "behavior modification" OR "cognitive behavioral therapy" OR "cognitive therapy" OR psychotherapy OR biofeedback OR neurofeedback OR "relaxation therapy" OR relaxation OR meditation OR mindfulness OR "mindfulness-based intervention*" OR "motivational interviewing" OR "stages of change" OR "group psychotherapy" OR "support group" OR "group counseling"

#5 #3 AND #4


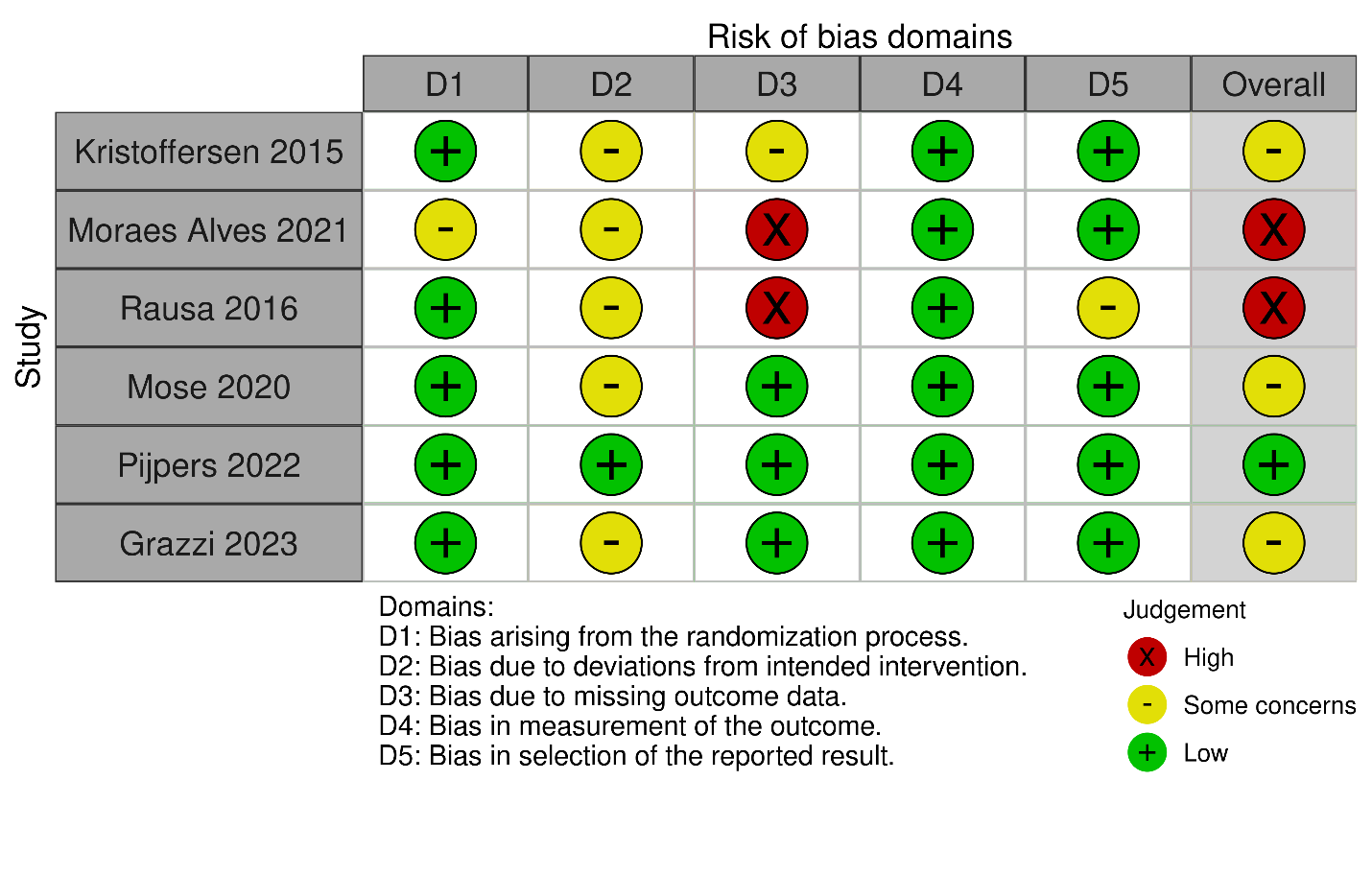


Supplemental Figure S1: Risk of bias assessment of the included studies

**Sensitivity analysis**


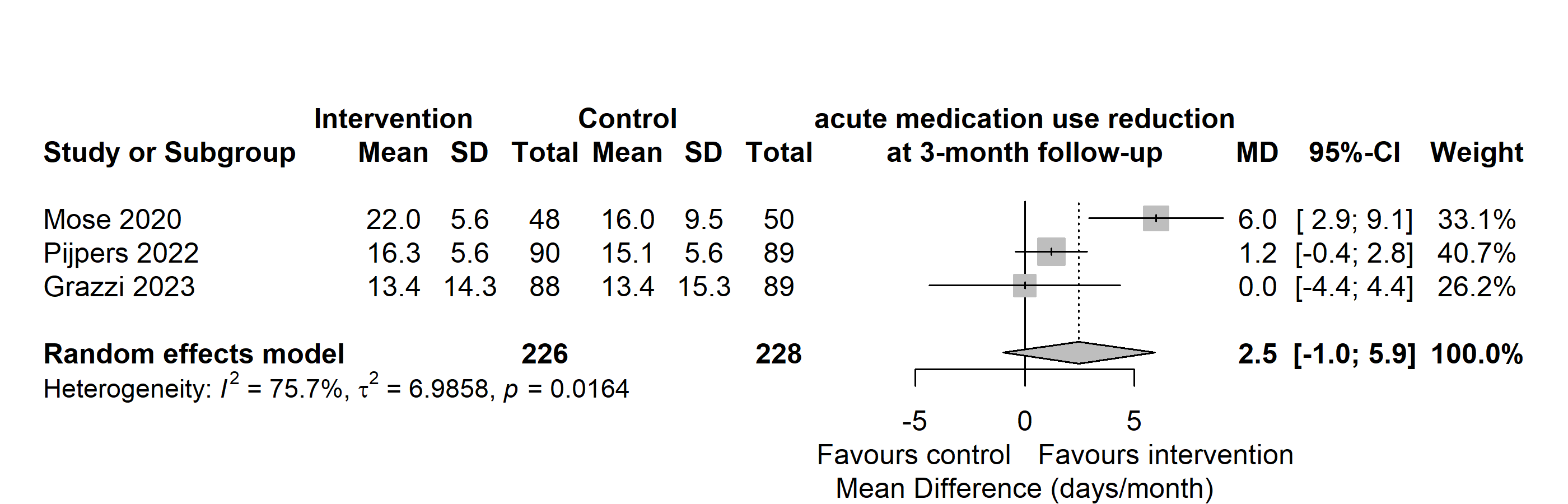


Supplemental Figure S2: Sensitivity analysis excluding a trial at high risk of bias: forest plot of the effect of interventions on acute medication use reduction at 3-month follow-up, compared with control.

*CI,* confidence interval; *I^2^ and τ^2^*, heterogeneity statistic; *MD,* mean difference; *SD,* standard deviation


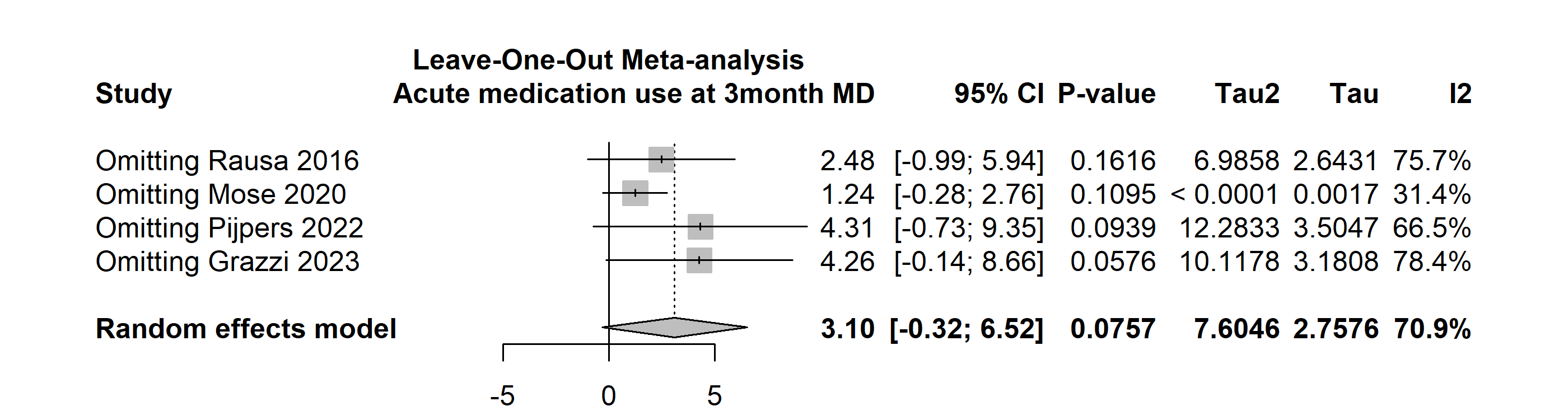

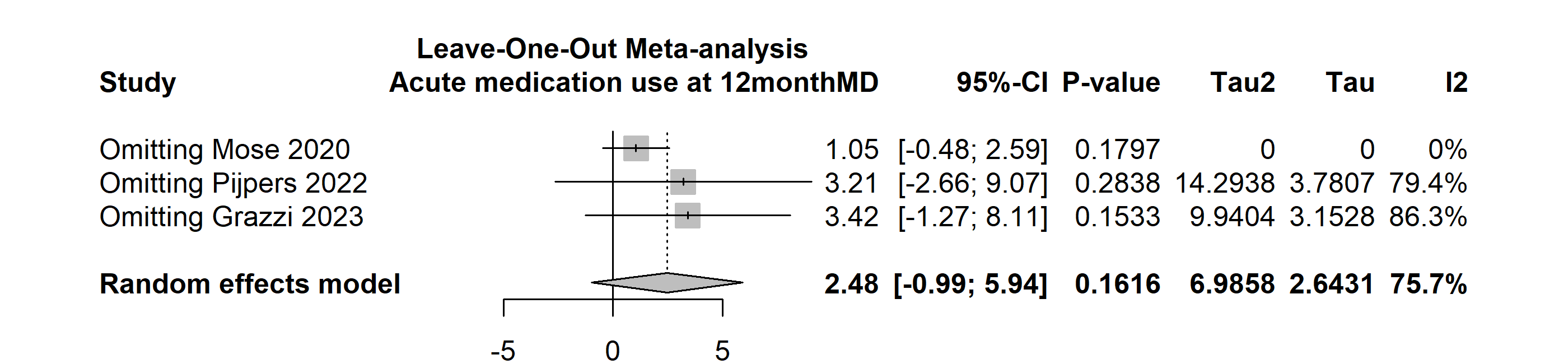


A.

B.

Supplemental Figure S3: Leave-one-out sensitivity analyses of the effect of interventions on acute medication use reduction at 3-month (A) and 12-month (B) follow-up.

*CI,* confidence interval; *I^2^ and Tau^2^*, heterogeneity statistic; *MD,* mean difference; *SD,* standard deviation


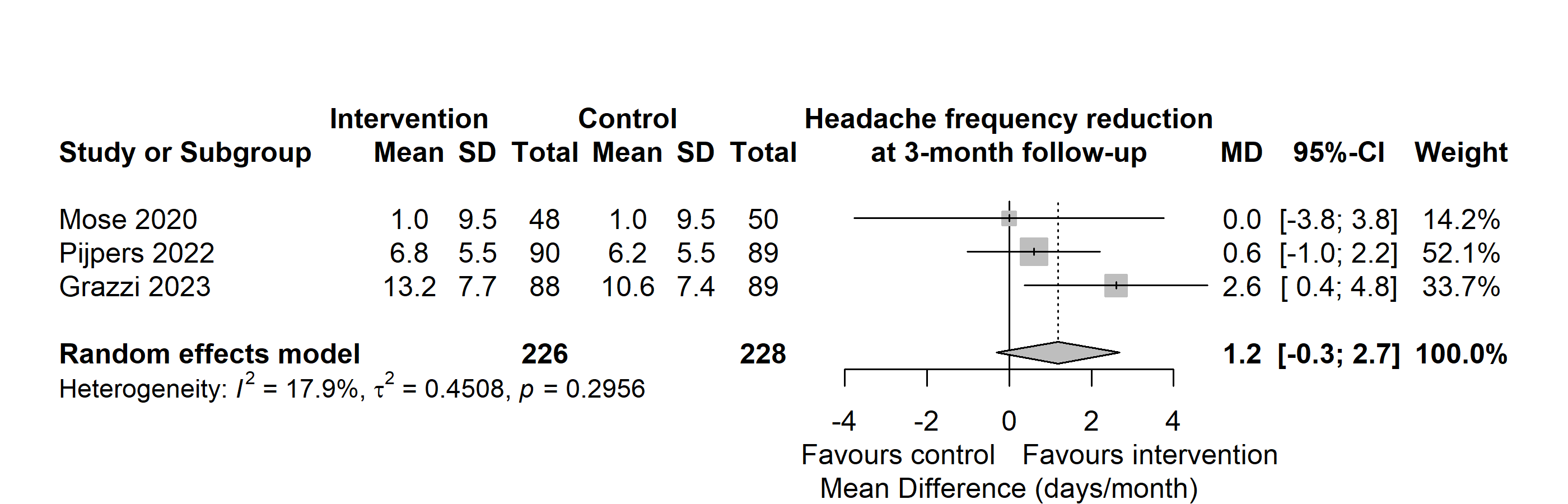


Supplemental Figure S4: Sensitivity analysis excluding trials at high risk of bias: forest plot of the effect of interventions on headache frequency reduction at 3-month follow-up, compared with control.

*CI,* confidence interval; *I^2^ and τ^2^*, heterogeneity statistic; *MD,* mean difference; *SD,* standard deviation


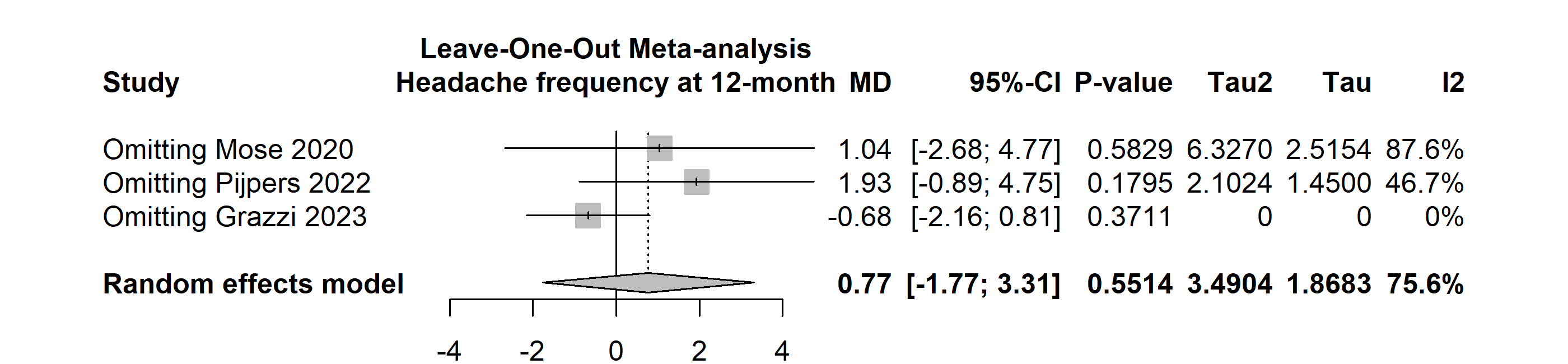


Supplemental Figure S5: Leave-one-out sensitivity analyses of the effect of interventions on headache frequency reduction at 12-month follow-up.

*CI,* confidence interval; *I^2^ and Tau^2^*, heterogeneity statistic; *MD,* mean difference; *SD,* standard deviation

| **Outcome** | **Studies** | **Participants** | **Effect** | **Downgrade reasons** | **Certainty** |
| --- | --- | --- | --- | --- | --- |
| Acute medication use, 3 months | 4 | 481 | MD 3.1 days/month,  95% CI -0.3 to 6.5 | Risk of bias, inconsistency, imprecision | Very low |
| Acute medication use, 12 months | 3 | 454 | MD 3.0 days/month,  95% CI -1.0 to 7.0 | Risk of bias, inconsistency, imprecision | Very low |
| Headache frequency, 3 months | 4 | 481 | MD 2.6 days/month,  95% CI -0.6 to 5.9 | Risk of bias, inconsistency, imprecision | Very low |
| Headache frequency, 12 months | 3 | 454 | MD 0.8 days/month,  95% CI -1.8 to 3.3 | Risk of bias, inconsistency, imprecision | Very low |

Supplemental Table S1: GRADE assessment of the certainty of evidence for acute medication use reduction and headache frequency reduction at 3- and 12-month follow-up.

*CI,* confidence interval; *MD,* mean difference
